# The Treatment of Acute Dysentery

**Published:** 1888-03

**Authors:** J. W. McLaughlin

**Affiliations:** Austin, Texas


DANIEL'S
### Texas Medical Journal.

PUBLISHED MONTHLY.—SUBSCRIPTION $2.00 A YEAR.

Vol. 3. AUSTIN, MARCH, 1888. No. 9.

By unanimous vote of the members, this Journal was made the Official Organ of the Austin District Medical Society.

### Original Articles.

CONTRIBUTED EXCLUSIVELY TO THIS JOURNAL.

*The Articles in this Department are accepted and published with the understanding that we are not responsible for, nor do we indorse the views and opinions of the writers by so doing.*

### THE TREATMENT OF ACUTE DYSENTERY.

*By J. W. McLaughlin, M. D., Austin, Texas.*

Read before Travis County Medical Society.

For Daniel's Texas Medical Journal.

IT is not my intention, nor do I believe it is your desire, that I should elaborately describe the various plans of treating this disease. Those who wish this information can obtain it in better shape from any of the numerous works on practical medicine.

The members of this society are selected according to rule to read papers upon selected subjects; the purpose of this plan, if I understand it, is to obtain the author's experience, to elicit discussion and thereby obtain an expression of views from the different members.

Acting upon this belief, I will confine my remarks to those plans of treating acute dysentery which have given the best results under my own observation.

It goes without saying that our treatment must be guided by our ideas regarding etiology, e. g., whether the disease in its origin is catarrhal, malarial or infectious, regarding its type whether sporadic, epidemic, sthenic or asthenic, and also by the condition of the patient with reference to his age, habits of life, previous history and his hygienic surroundings. For the relief of acute catarrhal dysentery, which is always sthenic in type, I rely principally upon salines and opium; these may be combined in such proportions and given in such vehicles as are best suited to the age, condition, or fancy of the patient; as adjuncts to this treatment, I recommend hot formentations to the bowels for the relief of tormina, and cocaine with morphine by the rectum for the relief of tenesmus.

If the disease should become sub-acute, that is, should linger beyond its time, and especially when the vital powers become much lowered, good results may be had from simaruba officinalis, with columba and opium.

In this stage, particularly if the inflammation has passed upward into the colon, or if ulceration of the bowels has occurred much benefit may be obtained from the use of large water injections. The water may be hot, warm or cold, whichsoever is most agreeable to the patient, and may be medicated by the addition of agents best suited to the nature of the case.

A favorite prescription which has done me excellent service is Labarraque's solution of chlorinated soda, 1 part to 40 of water, used in sufficient quantity to fill and distend the colon throughout. To accomplish this the patient's hips should be elevated above the plane of the body so that the fluid may pass from the syringe into the bowels, and by its own gravity be carried to the illeo cœcal valve. It is claimed that it can even force this valve and enter the small bowel.

Quinine is used in the malarial cases, and the mercuric salts in the so-called billious ones; pulv. ipecac in large doses will also benefit these, a few doses will quickly arrest the disease and restore the patient to health.

I have purposely used the term "so-called billious condition," for I seriously question if derangements of the liver cause this condition, and whether mercury posess any specific chologogue properties.

A patient with the following symptoms presents himself for treatment: Tongue heavily coated, breath very offensive, skin muddy and sallow; complains of languor, nausea, and want of appetite; in short, he has a typical case of "billiousness." We prescribe, say, 6 grains of calomel with 20 grains of sugar, divided into three powders, and direct that one powder be taken night and morning. The usual report will be that the first powder acted violently and caused large billious evacuations! the second did not act so freely and the third did not act at all. We find that his tongue has become clean, his breath is no longer offensive, his eyes and skin have cleared up, apetite returned and languor gone. I do not think any one, in the face of such evidence, would deny that the calomel has done this good work; many would say that it did this by stimulating the liver. This I cannot believe. I claim that it was not the liver but the intestinal follicles which the calomel stimulated, and that it was not the calomel but the pent up morbid secretion which caused the catharsis.

Now it is the duty of these intestinal follicles to eliminate certain waste products from the body. When they become torpid and fail to perform this work, these products of tissue waste will accumulate in the system and give rise to the so-called billious condition manifested in the color of the skin and eyes, the offensive breath, coated tongue, etc. Calomel acts directly—I believe almost specifically—upon these follicles, stimulates them to duty, whilst the quantity of secretion emptied through them into the bowels is the purgative agent for purgation is in direct proportion to the amount of secretion the bowel contains; hence the first powder acted profusely, the second less so and the third not at all. Ipecac acts in a similar manner; and it is in the malarial or billious varieties of dysentery that I have seen the best results from its use.

The diet in acute dysentery should be light und: unstimulating; it should consist probaby of that character of food which is mostly disposed of in the upper portion of alimentary tract and which leaves but little residue to pass over the inflamed lower bowel.

I labor under some difficulty in giving my treatment for infectious dysentery, inasmuch as the type of the disease and treatment indicated will vary with the nature of the infection.

The subject you selected for me to write upon is the "Treatment of Acute Dysentery," hence, I do not feel authorized to discuss its causes. Now these differ in their nature and in the results they produce ; any rational treatment must take cognisance of, and be guided by our opinion regarding the nature of this infection. The treatment indicated in that form of dysentery resulting from measles and many of the other infectious exanthemeta, is not the correct treatment for epidemic dysentery. Both forms of the disease are caused by contagion, but this greatly differs in the two varieties of dysentery in its nature, results, and in the means used for its control.

Perhaps the best way out of this difficulty is to pass unnoticed those types of dysentery which are frequently associated with the infectious exanthemeta. You will be benefitted by this plan, as it will considerably shorten my paper without entailing any serious loss, inasmuch as I usually treat these cases with slight modifications as I do those forms already passed upon.

Epidemic dysentery is always infectious, and as a rule is asthenic in type; this like other epidemic diseases may and often does prevail spasmodically. The nature of this infection is unknown, if it is caused by bacteria then the special form or forms which cause it have never been isolated, consequently the geimicide or toxic agents which would best destroy these microbes are also unknown. We are thus compelled to treat this disease symtomatically and rely upon those empirical remedies whch have been found by experience to do good work. Much will depend upon the doctors judgement and the proper application of remedies to meet particular indications. The following list of medicines comprise those upon which I usualiy rely to meet the various indications which come up in this form of dysentery: Opium, quinine, turpentine, camphor, napthaline, mercuric salts, chlorine water, nitrate silver, sulphurous acid, tannate bismuth, kino and ergot; concentrated food, meat preparations, milk and brandy. In those grave forms of dysentery characterized by profound toxæmic depression and threatened death from heart failure, I confidently recommend turpentine in large doses. It is an excellent hemostatic, a good antiseptic and a valuable stimulant, well suited from its prompt action to arrest the destructive tendency of this fearful malady, and counteract threatened heart failure. If the pulse is rapid and weak, the patient restless and sleepless, I would give morphia and chloral hydrate to secure rest and sleep; if the pulse is slow and sluggish, the patient inclined to stupor, would give small doses of atropine for its action upon terminal ends and ganglia of the vagus. Brandy, digitalis and strychina are indicated as cardiac stimulants and conservators, and ergot and kino to control intestinal hemorrhage. Quinine, mineral acids, carbo-ligin, resorcini and other antiseptics and astringents will be found useful in the varying phases of dysentery. I would also recommend injections of large quantities of water to which should be added antiseptics and astringents e. g. boric acid, borax and salicylic acid, tannic acid nitrate of silver. alum, or chlorine water. In the ordinary run of cases, those in which the system is less profoundly impressed and which pursue a slower pace, napthalin is recommended and in my hands has given excellent results. The mercuric salts and chlorine water are recommended in the diptherietic forms,sulphurous acid and tannate of bismuth in the typhoid forms and simaruba, columba, nux vomica and cinchona in the convalescent stages. Rest in bed, cleanliness of person and clothing with proper feeding are absolutely essential to the success of any plan of treatment. In this connection I wish to record my objection to that practice of stuffing patients, forcing upon them food when they neither desire it nor can digest it. I am certain that such practice is harmful to the patient. If the patient desires food and his stomach can digest it by all means let him have it, but when the contrary condition is present it is better to withhold food, or select that which is least objectionable to him; sweet milk, pancreatinized or with lime water; frozen cream if it is grateful, meat juice and meat broths are to be recommended. The same rule should govern us in giving whisky or brandy in dysentery, that are observed in its administration in other diseases. This, gentlemen, is an outline of the treatment which I usually adopt in the treatment of the various forms of acute dysentery; I submit it with the hope that it will elicit discussion and the society will thus get the benefit of each members experience in the treatment of this disease.

